# Study of bacterial respiratory infections and antimicrobial susceptibility profile among antibiotics naive outpatients visiting Meru teaching and referral hospital, Meru County, Kenya in 2018

**DOI:** 10.1186/s12866-023-02905-x

**Published:** 2023-06-29

**Authors:** Dinah Muthoni Miriti, John Maingi Muthini, Anthony Kebira Nyamache

**Affiliations:** Department of Biochemistry, Microbiology and Biotechnology, Kenyatta University, P.O Box 43844-00100, Nairobi, Kenya

**Keywords:** Prevalence, Antimicrobial susceptibility, Respiratory infections, Bacteria isolates, Antibiotics

## Abstract

**Objective:**

Respiratory tract infections cause significant morbidity and mortality globally and are the most common infectious diseases in humans. This study aims at assessing the presence of bacterial respiratory infections, number of people infected and antimicrobial susceptibility profile among antibiotic naïve outpatients presenting with respiratory tract infections in Meru Teaching and Referral Hospital.

**Methods:**

The study was conducted in Meru Teaching and Referral Hospital, Meru County from April 2017 to August 2018. Upper respiratory infections were characterized by acute infection of nasal cavity, pharynx and larynx while lower respiratory infections were characterized by chest pains, prolonged cough, productive sputum, difficulty in breathing, fever and weight loss. A total of 384 sputum and throat samples were collected aseptically from patients who were clinically suspected to have respiratory infections and cultured in blood agar, MacConkey agar and chocolate agar. Bacterial isolates were identified by colonial morphology, Gram stain and confirmed by biochemical tests. Antimicrobial susceptibility profile was determined using agar disc diffusion method.

**Results:**

Respiratory bacterial pathogens were isolated in 45.6% of the samples. The prevalence of the bacteria species isolated were as follows *Pseudomonas* species (36.6%), *Klebsiella* species (20.6%), *Staphylococcus aureus* (16.6%), *Streptococcus pyogenes* (13.7%), *Streptococcus pneumoniae (*10.3%) and mixed isolates (2.3%). Amoxicillin and ampicillin recorded the highest resistance rate. Most of the isolates displayed high level of resistance to more than two antibiotics. Although multidrug resistance is reported in the study, gentamicin, amikacin and cefuroxime are recommended as the antibiotics of choice against bacterial isolates obtained.

**Conclusion:**

Bacterial respiratory infections were prevalent in the study area and the isolates obtained showed resistance to commonly used antibiotics such as amoxicillin, ampicillin, ciprofloxacin piperacillin ciprofloxacin, ceftazidime, piperacillin-tazobactam and cephalexin. Therefore need for a continuous surveillance of antimicrobial resistance in management of respiratory infections in the study area.

## Introduction

Respiratory tract infections are termed as the infectious diseases of the respiratory tract and are the leading illnesses globally [1].These infections are classified as upper and lower respiratory tract infections and are the leading cause of morbidity and mortality especially in developing countries. Respiratory infections are the leading cause of heavy burden to public health [2]. In Kenya, these infections are considered as the major cause of morbidity [3]. Respiratory infections impose a serious financial burden to the economy due to loss of productivity and cost of antimicrobial agents prescribed by physician even when bacteria are not the main cause of respiratory infections [4].

The commonly known respiratory bacteria pathogens are *Staphylococcus aureus, Streptococcus pneumoniae*, *Pseudomonas* species, *Klebsiella* species, *Haemophilus influenzae.* Resistance to antibiotics is a global challenge to the health sectors especially in Kenya. This has been attributed to the emergence of mutant bacteria strains. Respiratory bacterial pathogens that are associated with reduced susceptibility to multiple classes of antibiotics include *Pseudomonas aeruginosa, Streptoococcus pneumoniae* and *Mycobacterium tuberculosis* [5]. Bacteria may acquire resistance to antibiotics through the following mechanisms; active efflux of the antibiotics, decreased permeability of the cell membrane, modification of drug target or inactivation of the antibiotics [6]. Factors that are attributed to the development of resistance bacteria include poor usage of antimicrobial agents, transmission of resistant bacteria among patients and from health care workers to patients and from patients to health care workers and poor guidelines in the administration of antimicrobial agents [7].

In developing countries such as Kenya, treatment of respiratory infections is done empirically and this may be due to expensive laboratory services and time as some of the tests takes time before the results are out. This has led to emergence of resistance bacterial pathogens in treatment of respiratory infections which is a serious problem in the health sector. Resistance to antimicrobial agents has led to therapeutical failure of empirical treatment [8]. Various research that have been done shows that a better understanding of the resistance mechanism in respiratory pathogens and correct diagnosis of the etiological agents of respiratory infections leads to better patient’s health, lowers morbidity, mortality and antimicrobial resistance [9].

Therefore, constant monitoring and surveillance of resistance pattern of respiratory bacterial pathogens to antimicrobial agents will not only guide the physician in the management of these infection but also helps in the evaluation of these infections [10]. This study was conducted to determine the presence of bacterial respiratory infections, number of people infected and antimicrobial susceptibility profile among antibiotic naïve outpatients in Meru Teaching and Referral Hospital. However, there are minimal reports on the presence of bacterial respiratory infections, number of people infected and the antimicrobial susceptibility pattern among outpatients presenting with respiratory tract infections in Meru Teaching and Referral, Meru County.

## Materials and methods

### Study design and study population

Hospital based cross sectional study was carried out between the month of April 2017 and August 2018. A total of 384 outpatients aged 5 years and above with symptoms of respiratory infections were randomly sampled from the outpatient department in Meru Teaching and Referral Hospital, Kenya as a representative of the population of interest.

### Inclusion criteria

Patients with respiratory clinical presentation who consented to participate in the study and those who had not taken antibiotics for a week prior to sampling.

### Exclusion criteria

Children aged below 5 years, outpatients who had taken antibiotics a week prior to sampling, all inpatients and those whose sputum smears were positive for acid fast bacilli were excluded from the study.

### Sample size and sampling technique

The sample size was determined using single proportion formula,

n = Z² P (1- P)

Ɛ²

Considering that z = 1.96 for 95% confidence interval corresponding to 5% significance level, n = estimated sample size while p = prevalence of the patients (if not known = 50%) and margin of error 5% (d = 0.05).

Therefore, n = (1.96)² x 0.5 (1- 0.5) = 384.

0.05²

Purposive sampling technique was used to select 384 participant for the study.

### Ethics approval and consent to participate

The study obtained ethical approval from Kenyatta University Ethics Review Committee (KUERC) (KU/R/COMM/51/470) and from the ethics committees of Meru Teaching and Referral Hospital (MRU/MED/GEN/R.14). Informed written consent was obtained from the participants. The study methods were carried out in accordance with relevant guidelines and regulations.

### Sample collection and laboratory processing

Sputum samples were collected into a clean wide mouthed container which was well labelled. All patients were guided on how to collect the sputum aseptically and take them to the laboratory immediately for analysis. Throat samples were collected using a sterile cotton swab moistened in saline water. This was done by a qualified medical officer. The samples were transported immediately into the microbiology laboratory at the hospital for analysis. Sputum samples that were viscous, mucoid or purulent were considered suitable for analysis. Gram stain smears were carried out for both samples and were examined microscopically. Ziehl Neelsen staining was done only for sputum samples so that the samples found to be positive for acid fast bacilli were not analysed.

### Bacterial isolation

Mucopurulent part of the sputum was inoculated onto sterile blood agar and MacConkey agar using streak method and incubated aerobically at 37 °C for 16 to 20 h. Throat swab samples were inoculated on blood agar and chocolate agar and then incubated in a candle jar at 37 °C for 24 to 48 h. Identification of bacterial isolates (*Pseudomonas* spp., *Klebsiella* spp., *Streptococcus pyogenes, Staphylococcus aureus* and *Streptococcus pneumoniae*) was done using colony morphology, colour, hemolysis on blood agar, Gram stain and various biochemical tests [11].

### Antimicrobial susceptibility testing

Antibiotic susceptibility testing was carried out using Kirby diffusion method on Muller Hinton agar (Oxoid) following Clinical and Laboratory Standard Institute recommendation [12]. Based on CLSI (2019) guidelines the antimicrobial agents used for Gram positive bacteria were; amoxicillin (30 µg), gentamicin (10 µg), cefuroxime (30 µg), ampicillin (10 µg), amikacin (30 µg), cephalexin (30 µg) and ceftazidime (30 µg) while for Gram negative bacteria were; cefuroxime (30 µg), amikacin (30 µg), piperacillin/tazobactam (110 µg), ampicillin (10 µg), gentamicin (10 µg), ciprofloxacin (5 µg), amoxicillin (30 µg) and ceftazidime (30 µg). The plates were incubated at 37 °C for 16 to 18 h and the diameters of the zone of inhibition were measured in millimeters. *Staphylococcus aureus* ATCC 25,923 was used as quality control for Gram positive bacteria while *Escherichia coli* ATCC 25,922 was used for Gram negative bacteria [13].

### Statistical analysis

Data collected was analysed using Statistical Package for Social Sciences version 25. The results obtained were presented in descriptive statistics using tables and percentages. Fisher’s exact test was used for statistical analysis of the positive cases according to patients’ characteristics. One way Analysis of variance (ANOVA) was used to determine statistical difference between mean of resistant Gram positive and Gram negative bacterial isolates to antibiotics. Statistical analysis was done at 95% confidence level and *P* < 0.05 was considered significant.

## Results

### Study subjects

Three hundred and eighty four sputum and throat swab samples were collected from outpatients who presented with symptoms of respiratory infections. Positive bacterial growth was recorded in 175/384 (45.6%) of the samples. Most of the bacterial pathogens were obtained from male patients 125 (71.4%) while female patients constituted only 50 (20.6%). The male to female ratio is 5:2 (Table 1).

**Table 1:** Demographic characteristics of patients with positive culture among antibiotic naïve patients visiting MTRH, 2018

Respiratory infections	Number	Positive cases	Prevalence (%)
Lower respiratory infection	264	132	45.5
Upper respiratory infection	120	43	35.8
**Sex**			
Male Female	204 180	125 50	61.3 27.8
**Total**	**384**	**175**	**45.6**

### Distribution of respiratory bacterial isolates

**Fig. 1:**
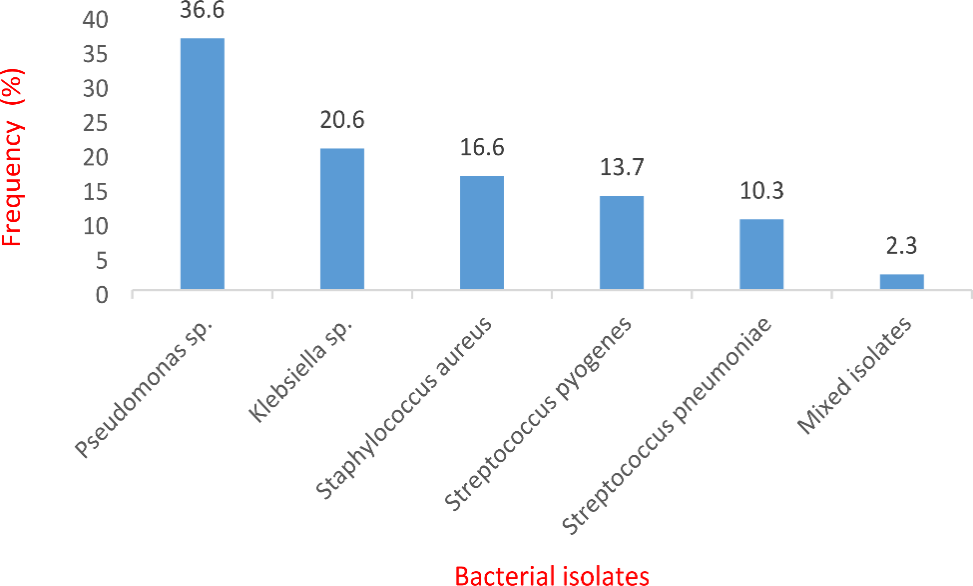
Frequency of bacterial isolates obtained from patients with respiratory infections

In this study, the Gram negative bacteria isolates obtained were 100 (57.1%) whereas the Gram positive isolates were 71 (40.6%). Only 4 (2.3%) bacterial isolates were mixed isolates. Five different bacterial isolates were isolated. These isolates include, *Pseudomonas* sp. 64 (36.6%), *Klebsiella* sp. 36 (20.6%), *Staphylococcus aureus* 29 (16.6%), *Streptococcus pyogenes* 24 (13.7%), *Streptococcus pneumoniae* 18 (10.3%) and mixed isolates 4 (2.3%) (Fig. 1). Both *Streptococcus pneumoniae* and *Staphylococcus aureus* were isolated from 3 sputum samples and *Streptococcus pyogenes* and *Staphylococcus aureus* from 1 sputum sample.

### Bacterial isolates obtained from the samples

Out of the 175 isolates, 125 bacterial isolates were obtained from sputum samples while 50 bacterial isolates from throat swabs. *Pseudomonas* species 64 (51.2%) were the predominant organism obtained from sputum samples followed by *Klebsiella* species 36 (28.8%), *Streptococcus pneumonia*e 11 (8.8%), *Staphylococcus aureus* 10 (8.0%) and 4 (3.2%) were mixed isolates (Table 2).

*Streptococcus pyogenes* 24 (48.0%) was the most common organism obtained from throat swab samples followed by *Staphylococcus aureus* 19 (38.0%) then *Streptococcus pneumoniae* 7 (14.0%) (Table 2).

**Table 2:** Bacterial isolates obtained from sputum and throat swab samples of patients with respiratory infections in MTRH

Sputum samples n = 125 Bacterial isolate	Number	Throat swab samples n = 50 Bacterial isolate	Number
*Pseudomonas* species	64 (51.2)	*Streptococcus pyogenes*	24 (48.0)
*Klebsiella* species	36 (28.8)	*Staphylococcus aureus*	19 (38.0)
*Streptococcus pneumoniae*	11 (8.8)	*Streptococcus pneumoniae*	07 (14.0)
*Staphylococcus aureus* Mixed isolates	10 (8.0) 04 (3.2)		

Key: *Values in parenthesis represent percentage of bacterial isolates

### Distribution of respiratory bacterial pathogens in different age groups and gender

The prevalence of isolated respiratory bacterial pathogens in patients in relation to their age group and gender were determined (Table 3). The patients’ age ranged from 5 years to 77 years. The mean age of the patients was 32 years, with a standard deviation of 5.64. Out of the 175 positive bacterial growth recorded, the percentage prevalence of isolates in different age groups ranged from 3.4 to 30.3%. The prevalence of bacterial respiratory infections in various age group is as follows; 5–14 years 25 (14.3%), 15–24 years 28 (16.0%), 25–34 years 53 (30.3%), 35–44 years 35 (20.0%), 45–54 years 16 (9.1%), 55–64 years 12 (6.9%) and above 65 years 6 (3.4%).

Mixed isolates were reported in 4 female patients, 3 in age group 25–34 years and 1 in age group 35–44 years.

In relation to gender, bacterial isolates were more predominant in males (71.4%) than in females (28.6%) (Table 3).

**Table 3:** Proportion of bacterial species isolated from patients with respiratory infection in different age groups and gender from patients with respiratory infections in MTRH

		Proportion of bacterial isolates n (%) Total (175)					
Age groups	*Pseudomonas* sp.	*Klebsiella* sp.	*S. pne*	*S. aur*	*S.pyog*	Mixed isolates	Total n (%)
5–14	10 (40.0)	4 (16.0)	2 (8.0)	6 (24.0)	3 (12.0)	0(0.0)	25 (14.3)
15–24	8 (28.6)	5 (17.9)	1 (4.0)	7 (25.0)	7 (25.0)	0 (0.0)	28 (16.0)
25–34	19 (35.8)	12 (22.6)	5 (9.4)	8 (15.1)	6 (11.3)	3 (5.7)	53(30.3)
35–44	10 (28.6)	9 (25.7)	7 (20.0)	5 (14.3)	3 (9.6)	1 (2.9)	35 (20.0)
45–54	7 (43.8)	3 (18.8)	3 (18.8)	1 (6.3)	2 (12.5)	0 (0.0)	16 (9.1)
55–64	6 (50.0)	3 (25.0)	0 (0.0)	1 (8.3)	2 (16.7)	0 (0.0)	12 (6.9)
Above 65	4 (66.7)	0 (0.0)	0 (0.0)	1 (16.7)	1 (16.7)	0 (0.0)	6 (3.4)
Total n (%)	64 (30.6)	36 (20.6)	18 (10.3)	29 (16.6)	24 (13.7)	4 (22.9)	175 (100.0)
Gender							
Male n = 125	41 (32.8)	28 (22.4)	09 (7.2)	25 (20.0)	22 (17.6)	0 (0.0)	
Female n = 50	10 (20.0)	03 (6.0)	15 (30.0)	07 (14.0)	11 (22.0)	4 (8.0)	

Key: (*S. pyog- Streptococcus pyogenes, S. aur- Staphylococcus aureus, S. pne- Streptococcus pneumoniae)*

### Antimicrobial resistance profile of the bacterial isolates

Antimicrobial agents that displayed the highest level of resistance were amoxicillin and ampicillin. High level of resistance was also recorded in ciprofloxacin, ceftazidime, piperacillin-tazobactam and cephalexin (Table 4). *Pseudomonas* spp. exhibited high level of resistance to most of the antibiotics used, cephalexin (100.0%), piperacillin-tazobactam (100.0%), ciprofloxacin (97.5%) and ceftazidime (80.0%). On the other hand it displayed low resistance levels in amikacin (0.0%), cefuroxime (6.0%) and gentamicin (7.0%).

*Klebsiella* spp. were more resistant to cephalexin (94.0%), ciprofloxacin (92.0%), piperacillin-tazobactam (84.0%) and ceftazidime (78.8%) but less resistant to gentamicin (0.0%), amikacin (2.0%) and cefuroxime (14.2%).

*Streptococcus pneumoniae* isolates were resistant to piperacillin-tazobactam (100.0%), cephalexin (80.0%), and ceftazidime (70.0%) but low resistance to gentamicin (0.0%), amikacin (6.0%) and cefuroxime (10.0%).

*Streptococcus pyogenes* isolates exhibited high resistance to ciprofloxacin (100.0%), ceftazidime (91.7%) and cephalexin (83.3%) and low resistance to gentamicin (0.0%), amikacin (0.0%) and cefuroxime (0.0%).

*Staphylococcus aureus* isolates were resistant to ciprofloxacin (92.0%), ceftazidime (89.0%) and piperacillin-tazobactam (67.0%) but lowest resistance to gentamicin (0.0%), amikacin (0.0%) and cefuroxime (0.0%). In mixed infections, isolates displayed 100.0% resistance to ciprofloxacin, ceftazidime and piperacillin-tazobactam.

**Table 4:** Antimicrobial resistance pattern of bacterial isolates obtained from antibiotic naïve outpatients with respiratory infections at MTRH, 2018

Antibiotics	Percentage of bacterial isolates resistant to antibiotics				
	*S. aureus* n = 29	* S.pyogenes* n = 24	* S.pneumoniae* n = 18	*Pseudomonas* sp. n = 64	*Klebsiella* sp. n = 36
Gentamicin	0.0	0.0	0.0	7.0	0.0
Cefuroxime	0.0	0.0	10.0	6.0	14.2
Amikacin	0.0	0.0	6.0	0.0	2.0
Cephalexin	-	83.3	80.0	100.0	94.0
Ceftazidime	89.0	91.7	70.0	80.0	78.8
Ciprofloxacin	92.0	100.0	-	97.5	92.0
Piperacillin/tazobactam Amoxicillin Ampicillin	67.0 100.0 100.0	- 97.6 100.0	100.0 100.0 100.0	100.0 100.0 100.0	84.0 100.0 100.0

Key: - not subjected to antibiotics

### Resistance of the bacterial isolates to the antimicrobial agents used

A total of 175 bacterial isolates were screened for antimicrobial resistance. Among the screened bacterial isolates, multidrug resistance was recorded in 81 (46.3%) of the isolates (Table 5). *Streptococcus pyogenes* is the only isolate that did not record multidrug resistance while *Pseudomonas* spp. showed highest level of multidrug resistance 59.4%.

**Table 5:** Multidrug resistance rate of the bacteria isolates among antibiotic naïve outpatients with respiratory infections at MTRH, 2018

Bacterial isolate	Total (%)	Pan susceptible	Multidrug resistant
*S. aureus*	29	13(44.8%)	16(55.1%)
*S.pyogenes*	24	24(100.0%)	0(0.0%)
*S.pneumoniae*	18	11(61.1%)	7(38.9%)
*Pseudomonas* sp.	64	26(40.6%)	38(59.4%)
*Klebsiella* sp. Mixed **Total**	36 04 **175**	16(44.4%) 04(100.0%) **94(53.7%)**	20(55.6%) 0(0.0%) **81(46.3%)**

## Discussion

The rate of bacterial respiratory infection, (45.6%) reported in this study was similar to a prevalence rate of 49.8% reported in Kenya [14] and 45.2% in Nigeria [15]. Although the rates of infections recorded in this study were higher than those previously obtained in Dadaab Camp in Kenya [16], Zarqa University [17] and in India [4], it was lower than those reported in Kibera, Nairobi (69.7%) [18] and in Pakistan (59.1%) [5]. The disparities in prevalence could be attributed to the differences in the study population, study period, socioeconomic status of the participant and geographical location. The current study sampled outpatients in the hospital while a study conducted in Nairobi, Kibera slum, Kenya sampled both outpatients and inpatients.

Bacterial growth were observed in 175(46.0%) samples of sputum and throat swab while 209 (54.0%) samples had no bacterial growth. This is contrary to a study conducted in Kenya that reported 95.4% growth of bacterial pathogens [19]. Previous studies carried out in Nepal reported no bacterial growth in 56.4% and 49.6% of the samples [20, 21]. The varying results in this study could be due to other etiological agents of respiratory infections which include viruses and fungi. The other reason could be that some patients did not follow the correct procedure during collection of sputum samples.

Among the bacteria isolates obtained, Gram negative bacteria were the highest accounting for 57.1% followed by Gram positive bacteria 40.6% and mixed infections 2.3%. These findings are contrary to a study done in Kenya that reported high prevalence of Gram positive bacteria [22]. However other related studies done elsewhere have reported a high prevalence of Gram negative bacteria [23–25]. From this study, more bacterial pathogens were isolated from sputum samples than throat swab. This was similar to a previous study in China [26] that reported more bacterial pathogens in cases of lower respiratory tract infections. The reason for this may be because sputum is more likely to be contaminated with upper respiratory tract secretions. The most common isolate was *Pseudomonas* species which was comparable to the observation documented in other studies [9, 19]. Previous studies done in Kenya have reported *Streptococcus pneumoniae* to be the most prevalent bacteria isolate [27, 28]. Other bacteria isolates obtained in this study include *Staphylococcus aureus* (22.2%), *Klebsiella* species (19.8%), *Streptococcus pyogenes* (14.8%), *Streptococcus pneumonia*e 12.3% and mixed isolates (2.3%). Similar results were reported in Nigeria [29]. The findings of this study are in contrast to those that were conducted in Nigeria and India [24, 30] where *Klebsiella* species was identified as the most prevalent isolate. This is because *Pseudomonas* species are widely found in the environment and spread easily through air droplets and contaminated hands of patients and health workers. The prevalence of 2.3% of mixed isolates obtained in the study was contrary to a related study carried out in Turkey that reported a prevalence of 11.2% of mixed isolates [31]. Among the mixed isolates, *Staphylococcus aureus* were the most common isolates. This was similar to a previous study in Iran that recorded *Staphylococcus aureus* as the most predominant isolate obtained from mixed pathogens [8].

Incidences of bacterial respiratory infections were more prevalent in age group 25–34 53(30.3%). A study conducted in Kenya reported higher cases of bacterial acute respiratory infections in age groups 17–50 years [23]. Similarly, a related study in Nigeria reported more cases of lower respiratory tract infections among patients aged between 21 and 40 years [32]. Age group above 65 years had the least number of infections. This was similar to a related study done in Kenya that reported low incidences of respiratory infections in patients aged above 56 years [5]. However, a study conducted in Pakistan reported more infections in age group above 60 years. The low rate of infections recorded in patients above 65 years could be due to the few number of patients in this age group who participated in the study. Patients above 65 years are elderly hence less mobile minimizing the chances of exposing them to risk factors of respiratory infections.

Similar to previous studies, majority of the patients were males accounting for 71.4% [5, 22]. This is contrary to previous related study in Kenya that reported higher cases of respiratory infections in females than in males [26]. This is explained by the high number of positive cultures obtained from male patients.

Resistance of respiratory bacterial pathogens to commonly used antibiotics has increased at an alarming rate making it difficult for clinicians to treat respiratory infections. This has led to emergence of infections caused by antimicrobial resistant bacteria associated with increased treatment failure, hospitalization, huge health budget and loss of life [10].

Among the tested antibiotics in the study, highest resistance rate was reported in amoxicillin and ampicillin. Resistance levels were also noted in other antibiotics such as ciprofloxacin, ceftazidime, piperacillin-tazobactam and cephalexin. A study conducted in Kenya reported a resistance prevalence of 72.0% to amoxicillin by Gram negative bacilli [32]. A study conducted in Iran reported resistance to the same class of antimicrobial agents [33]. Imani [34] reported a high level of microbial resistance to ampicillin. However, very low and in some cases no resistance rates were recorded for gentamicin, amikacin and cefuroxime. This observation concurs with previous studies conducted in India and Ethiopia that reported a high rate of susceptibility to gentamicin and cephalosporin especially cefuroxime [35, 36]. These antibiotics are administered intramuscular or intravenous injections hence they are less abused.

In this study, among the isolated pathogens, the Gram negative *Pseudomonas* species was found to be the most resistant isolate to most of the tested antimicrobial agents. This is similar to what was observed in Kenyatta National Hospital, Kenya [32]. The high developed antimicrobial resistance in *Pseudomonas* species could be attributed to its mechanisms of mutation and secretion of enzymes that inactivates the antibiotics.

Our study recorded MDR rate of 46.3% which lower than what has been reported previously in Ethiopian studies Arba Minch [9] and Jimma [37] 60.3% and 62.7% respectively. However it is higher than what was reported in South Ethiopia [38] 33.1%. The discrepancy may be due to use of different antibiotics and bacterial isolates. The high rate of resistance to tested antibiotics in our work is of great concern. This could be associated with indiscriminate use of the antimicrobial agents, community acquired resistance, inadequate infections control measures and spread of multidrug resistant isolates in the population [4]. *Pseudomonas* spp. was the isolate with the highest rate of MDR 59.4% followed by *Klebsiella* spp. 55.6%, *Staphylococcus aureus* 55.1% and *Streptococcus pneumoniae* 38.9%. A similar study in South Ethiopia reported MDR in *Staphylococcus aureus* 57.1%, *Klebsiella pneumoniae* 33.3% [38].

## Conclusion

The rate of bacterial respiratory infections among antibiotic naive outpatients visiting Meru Teaching and Referral Hospital is high, with commonly bacterial isolates associated with respiratory infections; *Pseudomonas* species, *Klebsiella* species, *Staphylococcus aureus, Streptococcus pyogenes*, and *Streptococcus pneumoniae.* Nevertheless, these organisms have developed antimicrobial resistance to at least more than two commonly used antibiotics like amoxicillin, ampicillin, ciprofloxacin, ceftazidime, piperacillin-tazobactam and cephalexin The results from this study shows high level of antibiotic resistance hence need for culture and susceptibility test for proper management of respiratory infections in the study area. This will curb the emergence and spread of resistance strains. The findings of this study will help the clinicians in the treatment and management of respiratory infections in Meru Teaching and Referral Hospital.

### Limitation

1. This study did not include the inpatients as the study population because majority of them were on antibiotics.
2. Most of the patients’ social demographic data required such as occupation, level of education, history of respiratory infections, smokers and alcoholism.

## Data Availability

The data used in this study is available from the corresponding author on request.

## List of abbreviations

ZN: Zeihl Neelsen
KUERC: Kenyatta University Ethics Review Committee
MDR: MultiDrug Resistant
MTRH: MeruTeaching and Referral Hospital
